# Adherence to the Mediterranean Diet Among Families from Four Countries in the Mediterranean Basin

**DOI:** 10.3390/nu17071157

**Published:** 2025-03-27

**Authors:** Ozge Yesildemir, Metin Guldas, Noemi Boqué, Lorena Calderón-Pérez, Perla Degli Innocenti, Francesca Scazzina, Nada Nehme, Fatima Abou Abbass, Marco de la Feld, Giuseppe Salvio, Nurcan Ozyazicioglu, Elif Yildiz, Ozan Gurbuz

**Affiliations:** 1 Department of Nutrition and Dietetics, Faculty of Health Sciences, Bursa Uludag University, 16059 Bursa, Türkiye; mguldas@uludag.edu.tr; 2 Department of Biotechnology, Graduate School of Natural and Applied Sciences, Bursa Uludag University, 16285 Bursa, Türkiye; ozang@uludag.edu.tr; 3 Technological Unit of Nutrition and Health, Eurecat, Technology Centre of Catalonia, 43204 Reus, Spain; noemi.boque@eurecat.org (N.B.); lorena.calderon@eurecat.org (L.C.-P.); 4 Human Nutrition Unit, Department of Food and Drug, University of Parma, 43125 Parma, Italy; perla.degliinnocenti@unipr.it (P.D.I.); francesca.scazzina@unipr.it (F.S.); 5 Department of Food Science and Technology, Faculty of Agricultural Engineering and Veterinary Medicine, Lebanese University, Dekwaneh 6573, Lebanon; nada.nehme@ul.edu.lb (N.N.); fatima.abouabbass.1@ul.edu.lb (F.A.A.); 6 ENCO Consulting, 80122 Naples, Italy; m.delafeld@enco-consulting.it (M.d.l.F.); salvio@enco-consulting.it (G.S.); 7 Department of Nursing, Faculty of Health Sciences, Bursa Uludag University, 16059 Bursa, Türkiye; nurcanoz@uludag.edu.tr; 8 Department of Food Engineering, Faculty of Agriculture, Bursa Uludag University, 16059 Bursa, Türkiye; elifyildiz@uludag.edu.tr

**Keywords:** Mediterranean diet, Mediterranean basin, multi-country study, family nutrition, dietary adherence, healthy eating habits

## Abstract

**Background/Objectives**: The cultural and socioeconomic changes to which societies are exposed can alter individuals’ lifestyles and dietary habits. The nutritional patterns of Mediterranean countries may also be affected by these factors at varying levels, depending on the changing social characteristics of the countries. This study aimed to determine Mediterranean diet (MedDiet) adherence among family members from four Mediterranean countries (Türkiye, Spain, Italy, and Lebanon). **Methods**: The survey was structured around sociodemographic data, family relationships, the Mediterranean diet adherence screener (MEDAS), the Mediterranean lifestyle index (MEDLIFE), the Mediterranean diet quality index (KIDMED), and the obstacles and drivers of MedDiet adherence. **Results**: The data were obtained from adults (n = 812), with the contribution of their children (n = 500) if they had any. According to the MEDAS, 22% of the adults strongly adhered to the MedDiet. Similar results were obtained when Mediterranean lifestyle adherence was analyzed using the MEDLIFE score, with 20% of the adults presenting a strong adherence, while significantly stronger adherence was found in Lebanon than in the other countries. Regarding the children and adolescents, around 30% presented strong adherence to the MedDiet. Price, availability, and accessibility were significant obstacles, whereas the perceptions of health and diet quality were identified as the drivers of the MedDiet. **Conclusions**: Overall, our results highlight the need for national policies to reduce the economic burden of healthy food options while encouraging equitable access to re-popularize the MedDiet pattern. Future research should focus on intervention strategies tailored to different age groups and socioeconomic backgrounds, the long-term impact of family-based strategies, and the cultural influences on MedDiet adherence.

## 1. Introduction

The Mediterranean diet (MedDiet) is more than just a dietary pattern. It also considers cultural and lifestyle factors, such as traditional cooking techniques, seasonal and local foods, color variety, conviviality, culinary practices, and physical activity [1]. Thanks to these features, the United Nations Educational, Scientific and Cultural Organization (UNESCO) recognizes this diet as intangible cultural heritage, representing a valid and certified dietary pattern for an optimal lifestyle [2]. Additionally, being a very diverse diet, it may vary from country to country in the Mediterranean basin, extending from Southern Europe to Northeast Africa. Observing the fundamental nutritional characteristics, the MedDiet is distinguished by a high consumption of olive oil, vegetables, fruits, whole grains, legumes, and nuts; a moderate intake of fish, seafood, eggs, and dairy products; and a low consumption of sweets and red and processed meat [3].

Long-term observational studies and randomized trials provide strong evidence that the MedDiet has many health benefits, including reducing the incidence of cardiovascular disease, obesity, hypertension, type 2 diabetes, metabolic syndrome, dyslipidemia, some cancers, and cognitive disorders [4]. Moreover, it is an eco-friendly option due to its small environmental footprint; it uses less water, energy, and land; and it emits fewer greenhouse gases [5]. However, despite this solid and increasing body of evidence, MedDiet adherence within populations from Mediterranean countries is alarmingly decreasing. Some studies have shown moderate to low-level MedDiet adherence in Mediterranean adults [6]. Regarding children and adolescents from Mediterranean countries, a recent study revealed that about half of the population had low-level adherence to this dietary pattern [7]. When looking at the specific countries included in this study—Türkiye, Spain, Italy, and Lebanon—current research highlights worrying trends. Some studies conducted in Türkiye have observed a significant decrease in adherence to the traditional MedDiet, particularly among young people [8,9]. Herrera-Ramos et al. (2023) also noted that MedDiet adherence has decreased among Spanish children and adolescents in the last 20 years due to deteriorating eating habits [10]. There is a notable decline in adherence to the MedDiet in Italy, particularly among younger people, primarily attributed to a reduction in olive oil consumption [11]. Lebanon, a country on the Mediterranean coast that has long been adherent to the MedDiet, is experiencing a shift in dietary choices away from it [12]. This has been related to lifestyle changes, along with cultural and socioeconomic modifications [13]. Mediterranean countries are undergoing a process of Westernization, whereby societies are drifting away from the healthy dietary patterns typical of Mediterranean populations [11]. The Western diet, indeed, is distinguished by a high consumption of processed and red meats, pre-packaged foods, high-sugar beverages, refined cereals, fried foods, high-fat dairy products, and high-fructose products [14]. Moreover, adherence to the MedDiet is influenced by additional factors, including problems related to the purchase, organization, preparation, and consumption of healthy food.

Family members could be the target population for re-establishing the MedDiet pattern in the Mediterranean basin. Moreover, identifying and addressing the factors influencing adherence to this food model are a crucial step in restructuring eating habits [15]. Given this context, studies on the factors affecting MedDiet adherence have recently been conducted among adults [16] and children/adolescents [7] in some Mediterranean countries. The impact of family dietary patterns on children’s eating habits is further highlighted by Rosi et al. (2024), who stated that family background characteristics may play a determinant role in MedDiet adherence [7]. However, to our knowledge, no previous study has explored adherence to the MedDiet of all family members together with the main barriers and facilitating factors. Therefore, this study aimed to determine the level of MedDiet adherence among family members from four Mediterranean countries (Türkiye, Spain, Italy, and Lebanon) and investigate the obstacles and drivers of this dietary pattern in the family context.

## 2. Materials and Methods

### 2.1. Participants

The current study was carried out in all four countries (Türkiye, Spain, Italy, and Lebanon) under the frame of the SWITCHtoHEALTHY project, which is part of the PRIMA Programme funded by the European Union under Grant Agreement number 2133. These countries were selected given their geographical coverage (from west to east to south of the Mediterranean basin) and as examples of countries with high malnutrition rates progressively abandoning a MedDiet style. The data for this cross-sectional observational study were collected using a survey addressed to adults (≥18 years) between July and October 2022. For a part of the survey, the involvement of children with the parents’ supervision was also suggested to investigate the eating habits of the children in the family (if any). All the procedures were carried out in accordance with the Declaration of Helsinki (1989) of the World Medical Association, and all the participants signed an informed consent form before they were enrolled in the study. The study protocol was approved by the Bursa Uludag University Ethics Committee, Türkiye (code: 2022-06; date of approval: 29 June 2022).

### 2.2. Data Collection

The data were collected in person or through an online, confidential survey filled out anonymously. The questionnaire was developed by using existing assessment tools, searching the current literature, and drawing on the research team’s expertise. The final questionnaire (Supplementary File S1) was translated into Turkish, Italian, Arabic, and Spanish. It was created using Google Forms and shared via word-of-mouth communication, social media, and personal connections to gather data.

### 2.3. Survey Structure

**Figure 1:**
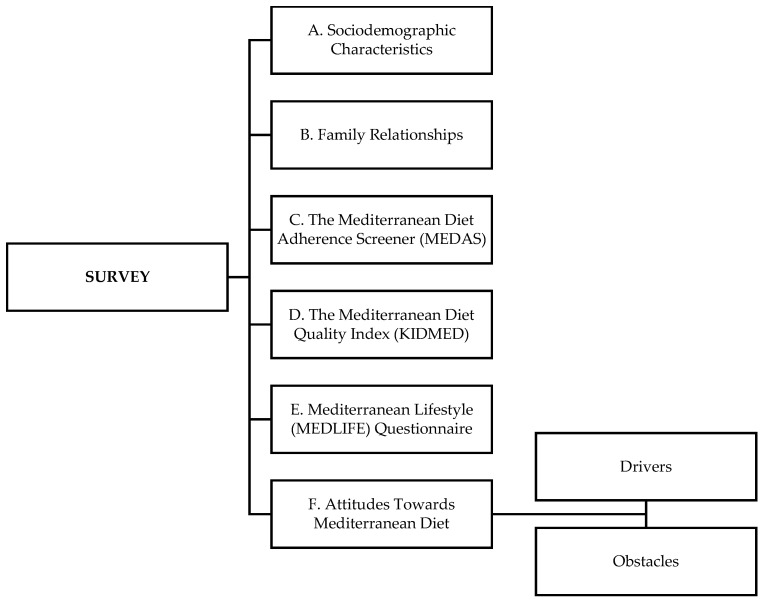
Organization chart of the survey.

The survey was structured to collect data on the following (Figure 1): (i) sociodemographics, (ii) family relationships, (iii) the Mediterranean diet adherence screener (MEDAS), (iv) the Mediterranean lifestyle (MEDLIFE) index, (v) the Mediterranean diet quality index (KIDMED), and (vi) obstacles to and drivers of adherence to the MedDiet.

#### 2.3.1. Sociodemographic Data

The first part of the survey included questions related to sociodemographic information about the participants, such as age, gender, education level, occupation, income status, marital status, health condition, type of family, and number of children. Individuals’ body weights and heights were also collected based on the respondents’ declarations. Body mass index (BMI) was determined by dividing body weight in kilograms by height in meters squared (m^2^). BMI was categorized according to the World Health Organization (WHO) classifications: underweight (<18.50 kg/m^2^), normal (18.50–24.99 kg/m^2^), overweight (25.00–29.99 kg/m^2^), and obese (≥30.00 kg/m^2^) [17].

#### 2.3.2. Family Relationships

This part of the survey included 8 questions related to family mealtime habits, such as having breakfast or dinner together or watching TV during meals.

#### 2.3.3. Mediterranean Diet Adherence Screener (MEDAS)

The validated 14-point MEDAS was used to measure adherence to the MedDiet. This tool was originally used in the PREDIMED (Prevención con Dieta Mediterránea) study [18]. It includes 12 questions regarding food frequency consumption and 2 questions about eating habits. Each answer is rated 0 or 1 depending on whether it is consistent with the Mediterranean diet. The ultimate score ranges from 0 to 14. A total score ≥7 marks acceptable adherence, whereas a score of ≥9 marks high adherence to the MedDiet [19].

#### 2.3.4. Mediterranean Lifestyle (MEDLIFE) Index

Sotos-Prieto et al. [20] developed the MEDLIFE index, a validated measure based on the Mediterranean Diet Pyramid principles promoted by the Spanish Mediterranean Diet Foundation. Its difference from MEDAS is that this one refers more to the MedDiet lifestyle, including items other than food intake. The MEDLIFE index includes 28 items, divided into 3 sections: (1) food consumption (15 items); (2) other dietary practices, such as coffee intake, consuming sugar-sweetened drinks, or sodium restriction (7 items); and (3) physical activity, rest, social habits, and conviviality (6 items). Each item is scored as 0 or 1. The final MEDLIFE index varies from 0 (worst adherence to the Mediterranean lifestyle) to 28 (highest adherence to the Mediterranean lifestyle) [20]. The scores are categorized into quartiles of adherence to the Mediterranean lifestyle: Quartile 1 (≤14 points), Quartile 2 (15–16 points), Quartile 3 (17–19 points), and Quartile 4 (≥20 points).

#### 2.3.5. Mediterranean Diet Quality Index (KIDMED)

KIDMED was created by Serra-Majem et al. in 2004 to evaluate children and adolescents’ adherence to the MedDiet. It has 16 yes/no questions, 4 of which address negative associations with the MedDiet (e.g., fast food, baked goods, sweets, and skipping breakfast), while the remaining 12 refer to positive associations (e.g., the consumption of oil, fish, fruits, vegetables, grains, nuts, legumes, pasta or rice, dairy products, and yogurt). A score of +1 or −1 may be attributed depending on whether the answer is relevant to the MedDiet. The total score ranges from 0 to 12. The KIDMED scores are divided into three categories: poor adherence (≤3 points), average adherence (4–7 points), and high adherence (≥8 points) [21]. The questions of KIDMED were answered only by parents with children aged 2–18 years. Each parent, therefore, filled in as many questionnaires as the number of children they had.

#### 2.3.6. Obstacles and Drivers to Mediterranean Diet Adherence

A questionnaire was developed to assess perceived obstacles to and drivers of MedDiet adherence through a review of the current literature [15,22,23]. A first list of possible drivers/obstacles was created based on this literature review, and, then, this list was refined to leave only 11 and 16 items, respectively (Table S1). The respondents scored each item on a 5-point Likert scale (1 = “not at all true for me” to 5 = “very true of me”). Five main themes about health, adherence, lifestyle, affordability, and environment were classified under drivers. Also, obstacles were categorized into three main themes: health, lifestyle, and affordability.

### 2.4. Statistical Analysis

Post hoc power analysis was conducted using G*Power (version 3.1.9.7, Universitat Düsseldorf, Düsseldorf, Germany) to assess the adequacy of the sample size. The effect size for the difference in MEDLIFE scores among the four countries was calculated as f = 0.27 (SD = 3.6). Based on this analysis, the study power (1 − β) was determined to be 99% at a statistical significance of 5%. The statistical data analysis was conducted using IBM SPSS Statistics Version 28 (IBM Inc., Armonk, NY, USA). The Shapiro–Wilk test was used to assess the data’s compliance with a normal distribution. Continuous variables are presented as arithmetic means (means) and standard deviations (SDs), while categorical variables are expressed as numbers (n) and percentages (%). The independent-sample t-test and one-way ANOVA were used with parametric data, while the Mann–Whitney U test and Kruskal–Wallis test were used for nonparametric data. The Bonferroni test was used as a multiple-comparison test. The differences between categorical variables were compared using Pearson’s chi-square and Fisher–Freeman–Halton tests. The Spearman correlation coefficients were calculated for the relationships between variables. The adherence percentages for each food and food group of the MEDAS items were determined, and the difference between the existing status and the ideal scenario (100% adherence) was depicted in a radar graph. Statistical significance was determined at *p* < 0.05.

## 3. Results

### 3.1. Subjects’ Characteristics

The characteristics of the sample population are shown in Table 1. In total, data were obtained from 812 adults: Türkiye (n = 201), Italy (n = 202), Lebanon (n = 209), and Spain (n = 200). The mean age of the adults was 41 ± 11 years. Mean age comparisons between countries revealed minor but significant variations (*p* < 0.001), with Spain having the oldest participants (46 ± 7 years), while the participants from Türkiye were the youngest (39 ± 9 years). Overall, the percentage of female participants was higher, especially in Italy (82.2%) and Türkiye (78.1%). Regarding the education level, one-third of the respondents were university graduates in all four countries, while regarding the occupation, almost 50% of the total population were office workers and service workers. Around 70% of the participants in all countries had a middle-income status, except for Lebanon, where this percentage corresponded to the low-income participants, being significantly higher than those in the other three countries (*p* < 0.001). The vast majority (83%) of the participants were married/with a partner. Almost all (96.1%) consisted of elementary families. About 40% of the total participants had two children. Regarding health status, 68% of the sample reported no diagnosed pathology. The mean BMI was 25.9 ± 4.8 kg/m^2^, significantly higher in Spain and Lebanon than in Italy (with a mean below 25 kg/m^2^), with intermediate values in Türkiye. In all the countries except Italy, the sample population comprised more than 40% overweight and obese individuals. At the country level, the rate of obese people was significantly higher in Lebanon and Spain than in Italy (*p* < 0.001). On the other hand, 11.3% of the participants reported having a food allergy, ranging from 7.2% in Lebanon to 16.3% in Italy, being intermediate in Türkiye. Finally, the mean sleep duration was 7 h, without differences between countries.

### 3.2. Adherence to Mediterranean Diet and Lifestyle

**Figure 2:**
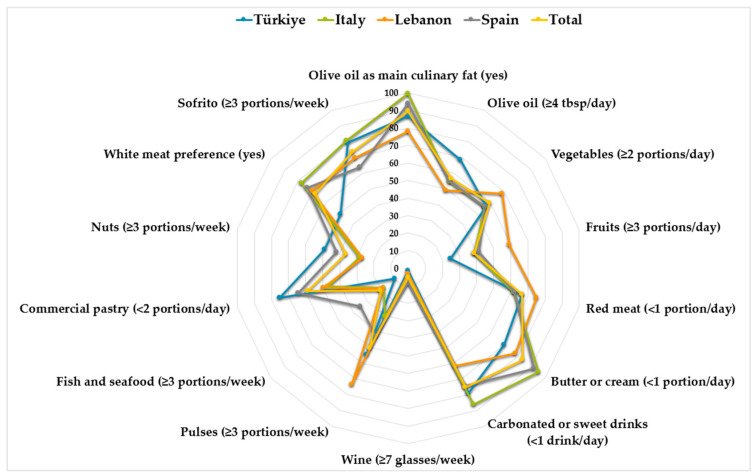
Radar graph of adherence to Mediterranean diet adherence screener (MEDAS) items regarding countries.

The participants’ MEDAS and MEDLIFE mean scores are provided in Table 2. The mean MEDAS score in the population was 7.82 ± 2.19, without differences between countries. Only 22.2% of the sample showed a high adherence to the MedDiet, while 26.8% reported a low adherence. Details about each MEDAS item are also included in Table 2 and displayed as a radar graph in Figure 2. The rate of people using olive oil as the main culinary fat was high, significantly greater in Italy than in the other three countries, at almost 100% of the sample, followed by Spain and Türkiye. However, only around 55% consumed ≥4 tablespoons of olive oil daily, significantly higher in Türkiye (69%) than in the other countries. Regarding vegetable consumption, 59% of the total sample stated eating at least two portions per day; in terms of the differences between countries, this proportion was significantly higher for Lebanese people (68.4%) than in other populations (*p* < 0.05). On the other hand, the consumption of fruits was generally deficient, with only 39% of the population reaching the intake of at least three portions per day, according to the MedDiet pattern. When comparing the four countries, the proportion of people with a positive score in this item was significantly lower in Türkiye (25%) and Italy (39%) than in Spain (41%) and Lebanon (59%) (*p* < 0.001). On the other hand, more than 60% of the participants consumed less than one portion of red meat every day, while white meat preference was lower (49%) in Turkish people than in others (more than 70%) (*p* < 0.001). The Italian and Spanish participants adhered more closely to a low consumption of butter, margarine, or cream than their Turkish and Lebanese counterparts (*p* < 0.001). In addition, 75% of all the respondents consumed one or fewer carbonated or sweet beverages daily, with the highest adherence detected in Italy (86.1%) and Türkiye (79%) (*p* < 0.001). Regarding wine, most stated that they did not consume ≥7 glasses/week. We found that the adherence to legume consumption (at least three portions per week) was significantly higher in Lebanon (73.7%), while Italy showed the lowest adherence (30.2%) (*p* < 0.001). The proportion of the population consuming at least three portions of fish or seafood per week was very low, ranging from 9.5% in Türkiye to 18% in Italy and Lebanon, and significantly higher in Spain (35%) (*p* < 0.001). Concerning the consumption of commercial pastries, half of our Italian and Lebanese populations consumed two or more portions daily, while this proportion was significantly lower in Spain and Türkiye (*p* < 0.001). Similarly, the percentage of the sample consuming less than three nuts per week, so being not aligned with this MedDiet principle, was significantly higher in Italy and Lebanon (around 73%) than in Spain and Türkiye, although it was more than 50% (*p* < 0.001). The proportions of the populations consuming ≥3 portions of sofrito weekly ranged from 64% to 80.7 for all the countries (Table 2, Figure 2).

Regarding adherence to the Mediterranean lifestyle, the mean MEDLIFE score across all the populations was 16.28 ± 3.68 (Table 2). There was a significant variation in the scores between countries (*p* < 0.001). Lebanon had a significantly higher mean score (17.95 ± 3.40) than the other countries, suggesting a higher adherence to the Mediterranean lifestyle. A high percentage of the sample fell into Quartile 1 (31.5%), meaning MEDLIFE scores of less than 14 points, indicating that a substantial proportion of individuals in Mediterranean countries presented a low adherence to the Mediterranean lifestyle. At the country level, Lebanese participants presented a significantly lower percentage in Quartile 1 (12.4%) and a higher percentage in Quartile 4 (34.0%) than the other populations.

Regarding MedDiet adherence among children and adolescents, the information provided by parents allowed for the collection of 124 KIDMED questionnaires in Türkiye, 101 in Italy, 124 in Lebanon, and 151 in Spain. The average KIDMED score was 6.18 ± 2.38, with no significant differences between countries (Table 2). About 30% of the population showed high adherence to the MedDiet, while more than half of the children/adolescents had average adherence to the MedDiet.

### 3.3. Family Relationships

The data on family relationships in the sample population are shown in Table S2. The percentage of families having breakfast together every day was quite low (27.2%), especially in Italy (20.8%) and Spain (20%). Many people (39.3%) also only had breakfast together on weekends. However, about 60% of families always had dinner together, which was higher than for breakfast. In addition, most households (more than 70%) had at least one parent accompany their children to dinner. Generally, 30% of the population watched television during family meals, but only 20% allowed their children to do the same. Most of the studied population never (17.2%) or occasionally (39.2%) answered the phone during family meals or allowed their children to respond (38.6% and 29.8%, respectively). Most families (46.6%) had guests in their homes every month.

As shown in Table S3, a statistically significant positive correlation was observed between the KIDMED score and the frequency of having breakfast together among all family members living at home (*r* = 0.093; *p* = 0.022). A negative relationship was also found between the KIDMED score and the parents’ frequency of watching television during family meals (*r* = −0.092; *p* = 0.025) and allowing their children to do the same (*r* = −0.141; *p* = 0.001).

### 3.4. Obstacles to and Drivers of Adherence to Mediterranean Diet

Detailed results about the obstacles to and drivers of adherence to the MedDiet for the sample population for each country are provided in Table S4. In the sample, the item ‘MedDiet contains healthier and more nutritious foods’ (4.37 ± 0.85) was scored with the highest rating regarding the drivers. Other highly rated drivers were as follows: MedDiet contains more healthy fats (4.29 ± 0.85), encourages higher consumption of fruits and vegetables and lower red meat consumption (4.26 ± 0.91), includes more unprocessed foods (4.22 ± 0.99), and increases consumption of homemade foods (4.21 ± 0.93). When comparing between countries, we found some differences, because the most highly rated driver of adherence to MedDiet in Türkiye was that it contains healthy fats, while in Italy, the most crucial driver of compliance with the MedDiet was that it includes more fruits and vegetables and less red meat (Table S4).

On the other hand, the highest-rated obstacle to adherence to the MedDiet was that ‘It includes high-priced foods’ (2.82 ± 1.28). The other barriers most highly rated as limiting adherence to the MedDiet are as follows: there are limited options in restaurants (2.49 ± 1.22), it is challenging to prepare meals suitable for the MedDiet (2.46 ± 1.28), it is not eligible for vegans (2.38 ± 1.33), and it contains allergenic foods (2.30 ±1.17). It was determined that cost was the most common obstacle to adherence to the MedDiet in Türkiye, Lebanon, and Spain. Conversely, the most important barrier in Italy was the need for more food variety in the MedDiet (Table S4).

Then, all the obstacles and drivers were grouped into different categories (health, diet quality, applicability, lifestyle, affordability, and environmental factors), and the results are shown in Table 3. We found significant disparities between countries in these categories under drivers. Health and diet quality showed significantly higher scores in Türkiye and Italy than the other populations, while Italy scored higher in applicability than Lebanon and Spain. Similarly, Italians rated items on lifestyle higher than Lebanese, with average scores among Spanish and Turkish people. Items about environmental factors were ordered with higher scores in Türkiye than in Lebanon and Spain. Among the barriers, we only found significant differences between countries regarding affordability. Specifically, Lebanese people rated these items on affordability higher than Italian and Turkish people. Generally, diet quality and health were identified as top-rated potential drivers for all populations. In contrast, all the participants rated affordability as a leading potential barrier.

## 4. Discussion

This study evaluated and compared the adherence to the MedDiet of four populations with distinct cultures and lifestyles but from the same geographic location, namely the Mediterranean basin. The results of this study show that families living in Mediterranean countries present a low-to-moderate adherence to the MedDiet and the Mediterranean lifestyle. Moreover, the differences found in the consumption of certain food groups revealed different socioeconomic, demographic, and lifestyle factors in these populations. The current research also investigated obstacles and drivers influencing the adoption of the MedDiet. Perception of health and diet quality were identified as major drivers. Financial concerns, poor availability, and accessibility were commonly noted obstacles to adherence to the MedDiet.

### 4.1. Adherence to Mediterranean Diet and Lifestyle

As the benefits of the MedDiet are increasingly recognized, many studies have examined adherence to it worldwide. A recent systematic review highlighted that Mediterranean populations seem to be shifting away from the MedDiet, with low and medium adherence ranging from 44.8% to 92.6% of adults in the included studies [24]. Biggi et al. (2024) reported medium-to-low adherence (88.9%) to the MedDiet in individuals from five Mediterranean countries [16], consistent with our results. The trend we observed in Türkiye, Italy, and Spain is comparable to other studies’ outcomes [8,9,25,26,27]. However, the MedDiet adherence of the Lebanese population was higher than that found in other studies [28,29]. The research exposed outcomes that could be associated with the economic crisis that Lebanon is currently experiencing [29]. Our study reflects the effects of the economic crisis because most of the Lebanese families participating in the survey reported low incomes. However, some changes caused by decreased individual purchasing power have favorably impacted MedDiet adherence. It is noted that the economic crisis has made it harder for Lebanese people to purchase red meat, butter, and sweets, and cooking with sofrito or vegetables has become increasingly popular [29]. This is also supported by the fact that the Lebanese adhered more to the Mediterranean lifestyle than the others, according to MEDLIFE, meaning that rest, social habits, and conviviality are in greater accordance with the Mediterranean lifestyle in Lebanon than in other countries.

Beyond the evaluation of MedDiet adherence, we also assessed the Mediterranean lifestyles using the MEDLIFE index, capturing adherence to an overall Mediterranean healthy lifestyle more holistically. To our knowledge, this is the first multi-country study on the Mediterranean lifestyle that, in addition to the MedDiet food patterns, assesses physical activity, rest, and social interactions such as socializing with friends or family. In the present study, a high percentage of our Mediterranean populations displayed low adherence to the Mediterranean lifestyle. This suggests that individuals living in Mediterranean countries are moving away not only from the consumption of MedDiet-specific foods but also from traditional healthy lifestyle habits and characteristics of the Mediterranean culture that could also be impacting the health status of these populations.

The most characteristic feature of the MedDiet is olive oil. In the present study, most of the participants indicated olive oil as the main culinary fat. This preference in all the countries could be due to its extensive production; it constitutes a dominant agro-industrial product [29]. Nearly 31% of olive oil production is in Italy, 28% in Spain, and 7.8% in Türkiye [30]. However, the amount of olive oil consumed daily by the participants was below the recommendations, which could be attributed to the fact that olive oil is more expensive than other types of vegetable oil. Although vegetables and fruits are more readily available and accessible in Mediterranean countries, their low consumption, especially fruits, in this study could be related to other factors like personal taste or social trends. The increasing access to a wide range of foods, including calorie-dense and processed options with a shift from traditional to Western-style diets [14], may have led people to prefer unhealthy and palatable foods instead of fruits and vegetables. The outcomes of our study also showed that most of the population consumed less than one portion of red meat per day. Although similar findings were formerly published in Türkiye [8], other investigations carried out in Spain and Italy showed more significant variability and lower rates [6,25,31]. The vast majority of the Lebanese participants in our study declared that they consumed one portion or less of red meat per day, suggesting that red meat consumption in the country has decreased even more compared to previous years [32,33]. The lower wine consumption observed in our study, especially in Muslim countries such as Türkiye and Lebanon, may be related to religious reasons. On the other hand, the higher consumption of legumes in Lebanon could be due to their low price, as discussed before [32]. Although fish is easily accessible throughout the year and is part of Mediterranean cuisine [34], only 20% of the respondents reached the recommended servings for fish consumption. Many reasons could explain this: its high price, a lack of knowledge about preparation techniques, a lack of preference due to the fish bones and fishy smell, a perceived lack of safety, and concerns about risks to human health due to environmental pollutants such as heavy metals [35]. Importantly, our findings pointed to a low intake of nuts in all the populations. These are similarly supported by previous data in Türkiye [8], Italy [36], and Spain [6]. This could be related to nuts being considered expensive. Additionally, people may be concerned about possible reactions to food allergens and gastrointestinal conditions that nuts could worsen [37].

It is suggested that younger people are more likely to have lower adherence to traditional dietary patterns compared to older ones [38]. A systematic review reported that MedDiet adherence is generally low to moderate among children and adolescents in the Mediterranean region [39]. Recent studies also declared that MedDiet adherence among young people has decreased further due to a shift toward Westernized dietary patterns, as well as disruptions to daily routines such as meal patterns and physical activity caused by quarantines and school closures during the COVID-19 pandemic [7,38,40]. The results of a population-based study showed a significant worsening of MedDiet adherence among Spanish children and adolescents, primarily due to increased consumption of refined foods, pastries, and fast foods, as well as a lower intake of fish, legumes, and fruits, in 2019–2020 compared to 1998–2000 [10]. Many studies have highlighted a noticeable decline in adherence to the MedDiet in Mediterranean countries, with high adherence ranging from 10% to 45% of children and adolescents in the countries involved in the current study [10,11,12,13,14,15,16,17,18,19,20,21,22,23,24,25,26,27,28,29,30,31,32,33,34,35,36,37,38,39,40,41,42,43,44,45,46]. A cross-sectional analysis of data from five Mediterranean countries (Italy, Spain, Portugal, Egypt, and Lebanon) reported that only about 40% of children and adolescents had high adherence to the MedDiet [7], which was even lower in our study. However, they showed that the population in Lebanon and Portugal was more compliant with the MedDiet than their counterparts in other countries, contrasting with our results, which found no difference between countries [38]. These findings suggest that adherence to the MedDiet may vary across Mediterranean populations and even within the same country.

### 4.2. Family Relationships

Family meals are an important aspect of social life in Mediterranean regions [47]. Family mealtimes, including the types of food served, parental modeling, and food socialization practices, have great potential to influence the eating behaviors of children [48]. Therefore, family meals may offer a promising entry point for the re-popularization of the MedDiet. However, with progressing globalization, urbanization, and modern lifestyles, fewer meals are being eaten together, resulting in the loss of the social component of the meal [47]. Families can be moved away from the traditional family meal structure due to different schedules, eating on the go, consuming ready-made meals, eating in front of digital tools, and eating separate meals simultaneously [48,49]. The current study supports the literature by finding that very few families have breakfast together daily, and a significant percentage do so only on weekends. Furthermore, our results revealed that dinner is a more shared meal than breakfast among family members, most likely because it is the only time of day when the whole family can gather, particularly on working days. These findings are consistent with previous research conducted in Spain, which found that the percentage of families who had breakfast together 5–7 days a week was 14.8%, but this rate increased to 76.9% for dinner [50]. Numerous studies also found that family breakfast occurs less often than family dinners [48,51].

For this reason, dinner is considered the most common shared meal as families eat together. Family meals have been highlighted as an important aspect of the home environment that promotes beneficial health behaviors in children and adolescents because of their routine nature [52,53]. The growing evidence suggests that having more frequent family meals is related to enhanced diet quality and improved food intake, including less consumption of sodium, sugar, fat, sweets, sugar-sweetened beverages, snacks, and fast food and a higher intake of dietary fiber, vitamins, minerals, dairy products, fruit, and vegetables in children and adolescents [48,54,55,56,57]. Our results, consistent with the literature, demonstrated the influence of sharing breakfast with the family on improving adherence to the MedDiet among children and adolescents. A study of Spanish adolescents indicated that, when the frequency of family breakfast increased, so did adherence to the MedDiet [50]. Italian children eating breakfast with their families showed higher KIDMED scores [58]. A recent study conducted with 2011 children and adolescents aged 6–17 from Mediterranean countries reported that eating with family members was related to higher MedDiet adherence [7]. The fact that having breakfast with the family is positively associated with MedDiet adherence among children and adolescents is significant because the new Mediterranean nutrition pyramid emphasizes that family meals are crucial. Surprisingly, we did not find an association for family dinners, demonstrating that breakfast is the most important meal in shaping the dietary patterns of children and adolescents. While family meals have been associated with healthy eating habits in children and adolescents, several factors might reduce the protective nature of family meals [59]. Distractions, such as watching television or using mobile devices, are among the leading factors [47]. Our findings suggest that the presence of television at family meals is linked to lower MedDiet adherence among children and adolescents. This result supports some prior studies showing a relationship between television viewing during family meals and lower overall diet quality in adolescents [59,60]. A systematic review found that watching television during family meals was associated with a higher intake of fried foods, pizza, sweets, snacks, and sugar-sweetened beverages and a negative association with consuming fruits and vegetables [61]. Moreover, a previous study observed that families without digital distractions during family meals had healthier dietary patterns [62]. This can be explained by the mindless eating hypothesis, which states that watching television during meals could lead to increased impulsive and unconscious consumption of food, particularly of more palatable and less satiating foods [63].

### 4.3. Obstacles to and Drivers of Adherence to Mediterranean Diet

To further understand and be able to design effective interventions to increase adherence to the MedDiet, it is crucial to identify the main factors that facilitate and hinder adherence to the MedDiet among Mediterranean populations. The findings of this research showed that attitudes toward health and diet quality are important drivers of adherence to the MedDiet in all the countries involved. Additionally, the participants identified drivers of the MedDiet as a reduced intake of red meat and increased consumption of vegetables, fruits, unprocessed foods, healthy fats, and homemade meals. Similarly, Scannell et al. [15] reported perceived health benefits and improved diet quality as the most important advantages influencing adherence to the MedDiet. Participants explicitly reported that adopting a MedDiet reduced red meat and saturated fat consumption, contributing to greater diet quality. Another study, conducted with 4025 people from Italy, Greece, Slovenia, Morocco, and Tunisia, indicated that favorable attitudes toward the healthiness of food were the biggest predictors of MedDiet adherence [16]. Perceptions of enhanced diet quality, including the consideration of naturalness, various health advantages, and good outcomes influencing physical health, body weight, and well-being, were determined as drivers of adherence to the MedDiet in a systematic review [22]. Additionally, Haigh et al. (2019) revealed that good nutrition knowledge and skills and an understanding of the links between diet and disease increase adherence to the MedDiet [64]. Therefore, supporting consumers’ knowledge about the benefits of MedDiet may improve their attitudes toward MedDiet adherence. It is also strongly recommended that intervention strategies be designed to raise consumers’ understanding of the health benefits of a balanced and nutritious diet.

Our results indicated that financial concerns, poor availability, and accessibility were commonly identified obstacles to adherence to the MedDiet in all populations. Various factors serve as obstacles to dietary changes. A systematic review of studies performed in four Mediterranean and eight non-Mediterranean countries highlighted eight obstacles to MedDiet adherence in adults. These included financial, cognitive, socio-cultural, motivational, lifestyle, accessibility and availability, sensory and hedonic, and demographic factors [22]. Economic concerns are frequently reported as obstacles to healthy eating [22,65]. In support of this, we found that high food costs linked to affordability were the most important obstacles to MedDiet adherence. It has been previously shown that a MedDiet is more expensive than a Western diet [66]. In a recent study, one-quarter of participants declared that high-priced foods made it more challenging to adhere to a MedDiet. There was a broad agreement that it was a costly dietary pattern, often emphasizing the high price of olive oil, vegetables, fruits, nuts, and fish [15]. The low cost of some typical foods of the MedDiet, like legumes, or potential substitutes for some foods to make an adapted MedDiet, like using locally accessible rapeseed oil and nuts, may offer solutions to these financial issues [22]. Moreover, consumers can be informed about seasonal products and directed to purchase these products. Markets where local products are sold can be expanded. Farmers can be supported economically, and domestic production can increase. In addition, seed and agricultural application studies can be carried out to increase food production suitable for the MedDiet. Another identified obstacle was the limited MedDiet options in restaurants. Similarly, a survey conducted in Mediterranean populations determined poor access, mainly restricted options in shops and restaurants, as a pertinent obstacle in Morocco and Slovenia [16]. It is highly recommended to suggest local food sources, tips for eating out, healthier restaurants, or menu options to overcome concerns about availability [67]. In addition, participants in our study stated that preparing meals suitable for the MedDiet was challenging. In this sense, Spanish research highlighted the lengthy food preparation time as an obstacle, especially for legumes [68]. Some ideas to overcome these concerns include the greater availability of pre-prepared foods, the promotion of straightforward recipes, and the encouragement of cooking classes and skill development [22]. There can be perceptions that some MedDiet foods are allergenic, unsuitable for vegans, or not healthy for patients with gastrointestinal diseases [15]. We also observed that some participants thought that the MedDiet was not suitable for vegans and that it contained allergenic foods. Therefore, promoting the health benefits of adhering to the MedDiet should be considered before adopting a dietary change [15]. However, in our study, the mean score of the drivers was about 4 points, while the mean score of the barriers was about 2 on a 5-point scoring system. Thus, drivers have a higher weight than barriers regarding adherence to the MedDiet in our population.

Our study has several limitations. First, the sample size is relatively limited, which may restrict the generalizability of the findings. Future studies with larger sample sizes could enhance the robustness and applicability of the results. Additionally, the data rely on self-reported information from participants. This may have introduced recall bias and the possibility of socially desirable responses, particularly regarding body weight and dietary habits. Future research could incorporate more objective assessment methods to reduce these biases. Another limitation of this study is its cross-sectional design, which makes it difficult to establish causal relationships. Longitudinal studies would help us to better understand how MedDiet adherence changes over time. Moreover, although MEDAS, MEDLIFE, and KIDMED are the most frequently used tools for assessing adherence to the MedDiet, they may not fully capture any unmeasured important components of this dietary pattern, causing the adherence rate to be underestimated or overestimated. Alternative dietary assessment methods could be employed for a more in-depth analysis of food consumption habits. Finally, using social media to recruit participants may have introduced selection bias by favoring individuals with higher social media engagement. This approach may also have limited the diversity of the sample, as those without access to or interest in social media platforms may have been underrepresented. These factors should be considered when interpreting the findings. Despite these limitations, this study provides valuable insights into the factors affecting MedDiet adherence and offers important findings that can guide future research.

## 5. Conclusions

The current study, conducted in four Mediterranean countries, determined that, in general, the adherence to the MedDiet and the Mediterranean lifestyle among families was intermediate, with a low proportion of adults, children, and adolescents showing high adherence to the MedDiet, and with variations in eating patterns between countries. Family meals, like having breakfast together, were identified as an important target for increasing adherence to the MedDiet by children and adolescents. In this context, families should be targeted in designing strategies to increase MedDiet adherence. Health and diet quality were identified as the most relevant drivers of adherence to the MedDiet, while the most critical obstacle was financial concerns about maintaining the MedDiet pattern. This suggests that policies focused on reducing the economic burden of healthy food options and promoting equitable access are crucial. Expanding markets for local products, economically supporting farmers, increasing domestic production, and encouraging the consumption of seasonal foods can further enhance adherence, ultimately unlocking the potential of the MedDiet to improve public health outcomes and support lasting sustainability globally. The conclusions of the present study could help us to design interventions focused on increasing adherence to the MedDiet. Future research should explore effective intervention strategies tailored to different age groups and socioeconomic backgrounds to enhance MedDiet adherence. Additionally, longitudinal interventional studies assessing the long-term health impact of family-based dietary interventions focused on increasing MedDiet adherence could provide valuable insights. Further investigations are also needed to evaluate the role of digital tools and educational programs in promoting MedDiet adherence among younger populations. Moreover, we also recommend implementing large-scale studies of MedDiet adherence in African and Middle Eastern Mediterranean countries. Lastly, examining the potential interactions between cultural influences and dietary habits across Mediterranean countries may help us to refine targeted public health strategies.

## Figures and Tables

**Table 1:** Characteristics of the sample population.

	Türkiye (n = 201)	Italy (n = 202)	Lebanon (n = 209)	Spain (n = 200)	Total (n = 812)	*p*
Age of adults (years), mean ± SD	39.1 ± 8.6 ^a^	42.1 ± 10.4 ^b^	42.9 ± 13.2 ^b^	46.1 ± 7.1 ^c^	41 ± 11.1	**<0.001**
Gender, n (%)						
Female	157 (78.1) ^a^	166 (82.2) ^a^	104 (49.8) ^b^	108 (54.0) ^b^	535 (65.9)	**<0.001**
Male	44 (21.9)	36 (17.8)	105 (50.2)	92 (46.0)	277 (34.1)	
Educational status, n (%)						
Non-finished primary school	2 (1.0) ^a^	1 (0.5) ^a^	17 (8.1) ^b^	4 (2.0)^a^	24 (3.0)	**<0.001**
Primary school	10 (5.0) ^a^	0 (0.0) ^b^	24 (11.5) ^a^	22 (11.0) ^a^	56 (6.9)	
Secondary school	10 (5.0) ^a^	12 (5.9) ^a^	38 (18.2) ^b^	9 (4.5) ^a^	69 (8.5)	
High school	36 (17.9) ^ab^	52 (25.7) ^b^	30 (14.4) ^a^	30 (15.0) ^a^	148 (18.2)	
Vocational school	15 (7.5) ^a^	14 (6.9) ^a^	20 (9.6) ^a^	57 (28.5) ^b^	106 (13.1)	
University	69 (34.3)	70 (34.7)	74 (35.4)	62 (31.0)	275 (33.9)	
Post-graduate	59 (29.4) ^a^	53 (26.2) ^a^	6 (2.9) ^b^	16 (8.0) ^b^	134 (16.5)	
Occupation, n (%)						
Manager	23 (11.4) ^a^	24 (11.9) ^a^	9 (4.3) ^b^	12 (6.0) ^ab^	68 (8.4)	**<0.001**
Academic laborer	62 (30.8) ^ab^	6 (3.0) ^a^	26 (12.4) ^c^	12 (6.0) ^bc^	106 (13.1)	
Office worker	36 (17.9) ^ab^	88 (43.6) ^c^	31 (14.8) ^b^	55 (27.5) ^a^	210 (25.9)	
Service worker	44 (21.9) ^a^	16 (7.9) ^b^	85 (40.7) ^c^	42 (21.0) ^a^	187 (23.0)	
Agriculture and forestry worker	1 (0.5) ^a^	5 (2.5) ^b^	9 (4.3) ^a^	9 (4.5) ^a^	24 (3.0)	
Blue-collar worker	5 (2.5) ^a^	28 (13.9) ^bc^	49 (23.4) ^c^	15 (7.5) ^ab^	97 (11.9)	
Engine worker	11 (5.5) ^a^	7 (3.5) ^a^	0 (0.0) ^b^	16 (8.0) ^a^	34 (4.2)	
Healthcare worker	15 (7.5) ^a^	25 (12.4) ^a^	0 (0.0) ^b^	21 (10.5) ^a^	61 (7.5)	
Unskilled laborer	4 (2.0) ^a^	3 (1.5) ^a^	0 (0.0) ^a^	18 (9.0) ^b^	25 (3.1)	
Income status, n (%)						
Low	17 (8.5) ^a^	19 (9.4) ^a^	144 (68.9) ^b^	24 (12.0) ^a^	204 (25.1)	**<0.001**
Middle	143 (71.1) ^a^	141 (69.8) ^a^	56 (26.8) ^b^	146 (73.0) ^a^	486 (59.9)	
High	41 (20.4) ^a^	42 (20.8) ^a^	9 (4.3) ^b^	30 (15.0) ^a^	122 (15.0)	
Marital status, n (%)						
Single	40 (19.9) ^a^	35 (17.3) ^a^	35 (16.7) ^a^	0 (0.0) ^b^	110 (13.5)	**<0.001**
Separated/divorced	16 (8.0) ^a^	7 (3.5) ^a^	5 (2.4) ^ab^	0 (0.0) ^b^	28 (3.4)	
Married/with partners	145 (72.1) ^a^	160 (79.2) ^a^	169 (80.9) ^ab^	200 (100.0) ^b^	674 (83.0)	
Type of family, n (%)						
Elementary family	190 (94.5) ^ab^	197 (97.5) ^ab^	195 (93.3) ^b^	198 (99.0) ^a^	780 (96.1)	**0.011**
Extended family	11 (5.5)	5 (2.5)	14 (6.7)	2 (1.0)	32 (3.9)	
Disease status, n (%)						
Non declared pathology	134 (66.7)	141 (69.8)	129 (61.7)	148 (74.0)	552 (68.0)	0.056
Declared pathology	67 (33.3)	61 (30.2)	80 (38.3)	52 (26.0)	260 (32.0)	
BMI (kg/m^2^), mean ± SD	24.9 ± 4.3 ^ab^	23.9± 4.1 ^a^	26.5 ± 4.5 ^b^	25.9 ± 4.7 ^b^	25.9 ± 4.8	**<0.001**
BMI classification, n (%)						
Underweight	3 (1.5)	8 (4.0)	1 (0.5)	5 (2.5)	17 (2.1)	**<0.001**
Normal	113 (56.2) ^ab^	125 (61.9) ^b^	88 (42.1) ^c^	95 (47.5) ^ac^	421 (51.8)	
Overweight	57 (28.4) ^ab^	55 (27.2) ^b^	83 (39.7) ^a^	67 (33.5) ^ab^	262 (32.3)	
Obese	28 (13.9) ^ab^	14 (6.9) ^b^	37 (17.7) ^a^	33 (16.5) ^a^	112 (13.8)	
Presence of food allergy, n (%)						
Yes	16 (8.0) ^ab^	33 (16.3) ^b^	15 (7.2) ^a^	28 (14.0) ^ab^	92 (11.3)	**0.006**
No	185 (92.0)	169 (83.7)	194 (92.8)	172 (86.0)	720 (88.7)	
Sleep duration, mean ± SD	7.0 ± 1.2	6.9 ± 1.0	6.6 ± 1.5	6.8 ± 1.2	6.9 ± 1.3	0.060
Number of children, n (%)						
None	55 (27.4)	61(30.2)	51 (24.4)	49 (24.5)	216 (26.6)	**<0.001**
One	63 (31.3) ^a^	40 (19.8) ^b^	32 (15.3) ^b^	5 (2.5) ^c^	140 (17.2)	
Two	67 (33.3) ^a^	79 (39.1) ^a^	62 (29.7) ^a^	106 (53.0) ^b^	314 (38.7)	
≥Three	16 (8.0) ^a^	22 (10.9) ^ab^	64 (30.6) ^c^	40 (20.0) ^bc^	142 (17.5)	
Age of children (years), mean ± SD	13.4 ± 7.8 ^a^	12.5 ± 5.6 ^a^	14.6 ± 7.8 ^a^	11.2 ± 4.7 ^b^	12.4 ± 6.7	**<0.001**

BMI: Body mass index. Kruskal–Wallis test. Chi-square test. The ‘a’, ‘b’, and ‘c’ superscripts show the results of pairwise comparisons between countries; values with different letters were significantly different among groups. The bold values indicate statistically significant values (*p* < 0.05).

**Table 2:** Adherence to the Mediterranean diet of the sample population.

For Adults	Türkiye (n = 201)	Italy (n = 202)	Lebanon (n = 209)	Spain (n = 200)	Total (n = 812)	*p*
**MEDAS score, mean ± SD**	7.71 ± 2.05	7.71 ± 2.07	7.82 ± 1.98	200	7.82 ± 2.19	0.353
**Level of adherence to the MedDiet, n (%)**						
Low	56 (27.9)	55 (27.2)	54 (25.8)	53 (28.0)	218 (26.8)	0.359
Acceptable	107 (53.2)	108 (53.5)	108 (51.7)	91 (45.5)	414 (51.0)	
High	38 (18.9)	39 (19.3)	47 (22.5)	56 (28)	180 (22.2)	
**MEDAS Items**						
**Olive oil as main culinary fat, n (%)**						
Yes	174 (86.6) ^ab^	201 (99.5) ^c^	163 (78.0) ^b^	188 (94.0) ^a^	726 (89.4)	**<0.001**
No	27 (13.4)	1 (0.5)	46 (22.0)	12 (6.0)	86 (10.6)	
**Olive oil (≥4 tbsp/day), n (%)**						
Yes	138 (68.7) ^a^	110 (54.5) ^b^	103 (49.3) ^b^	110 (55.0) ^b^	461 (56.8)	**<0.001**
No	63 (31.3)	92 (45.5)	106 (50.7)	90 (45.0)	351 (43.2)	
**Vegetables (≥2 portions/day), n (%)**						
Yes	114 (56.7) ^a^	113 (55.9) ^a^	143 (68.4) ^b^	112 (56.0) ^a^	482 (59.4)	**0.022**
No	87 (43.3)	89 (44.1)	66 (31.6)	88 (44.0)	330 (40.6)	
**Fruits (≥3 portions/day), n (%)**						
Yes	50 (24.9) ^a^	58 (38.7) ^a^	124 (59.3) ^b^	83 (41.5) ^c^	315 (38.8)	**<0.001**
No	151 (75.1)	144 (71.3)	85 (40.7)	117 (58.5)	497 (61.2)	
**Red meat (<1 portion/day), n (%)**						
Yes	134 (66.7) ^abc^	125 (61.9) ^c^	157 (75.1) ^b^	125 (62.5) ^ac^	541 (66.6)	**0.016**
No	67 (33.3)	77 (38.1)	52 (24.9)	75 (37.5)	271 (33.4)	
**Butter or cream (<1 portion/day), n (%)**						
Yes	141 (70.1) ^a^	192 (95.0) ^b^	163 (78.0) ^a^	183 (91.5) ^b^	679 (83.6)	**<0.001**
No	60 (29.9)	10 (5.0)	46 (22.0)	17 (8.5)	133 (16.4)	
**Carbonated or sweet drinks (<1 drink/day), n (%)**						
Yes	159 (79.1) ^ab^	174 (86.1) ^b^	129 (61.7) ^c^	149 (74.5) ^a^	611 (75.2)	**<0.001**
No	42 (20.9)	28 (13.9)	80 (38.3)	51 (25.5)	201 (24.8)	
**Wine (≥7 glasses/week), n (%)**						
Yes	3 (1.5) ^a^	15 (7.4) ^b^	7 (3.3) ^ab^	16 (8.0) ^b^	41 (5.0)	**0.006**
No	198 (98.5)	187 (92.6)	202 (96.7)	184 (72.0)	771 (95.0)	
**Legumes (≥3 portions/week), n (%)**						
Yes	110 (54.7) ^a^	61 (30.2) ^b^	154 (73.7) ^c^	86 (43.0) ^a^	411 (50.6)	**<0.001**
No	91 (45.3)	141 (69.8)	55 (26.3)	114 (57.0)	401 (49.4)	
**Fish and seafood (≥3 portions/week), n (%)**						
Yes	19 (9.5) ^a^	36 (17.8) ^a^	38 (18.2) ^a^	70 (35.0) ^b^	163 (20.1)	**<0.001**
No	182 (90.5)	166 (82.2)	171 (81.8)	130 (65.0)	649 (79.9)	
**Commercial pastry (<2 portions/day), n (%)**						
Yes	151 (75.1) ^a^	95 (47.0) ^b^	104 (49.8) ^b^	129 (64.5) ^a^	479 (59.0)	**<0.001**
No	50 (24.9)	107 (53.0)	105 (50.2)	71 (35.5)	333 (41.0)	
**Nuts (≥3 portions/week), n (%)**						
Yes	98 (48.8) ^a^	57 (28.2) ^b^	56 (26.8) ^b^	84 (42.0) ^a^	295 (36.3)	**<0.001**
No	103 (51.2)	145 (71.8)	153 (73.2)	116 (58.0)	517 (63.7)	
**White meat preference, n (%)**						
Yes	99 (49.3) ^a^	157 (77.7) ^b^	148 (70.8) ^b^	147 (73.5) ^b^	551 (67.9)	**<0.001**
No	102 (50.7)	45 (22.3)	61 (29.2)	53 (26.5)	261 (32.1)	
**Sofrito (≥3 portions/week), n (%)**						
Yes	160 (79.6) ^a^	163 (80.7) ^a^	146 (69.9) ^ab^	128 (64.0) ^b^	597 (73.5)	**<0.001**
No	41 (20.4)	39 (19.3)	63 (30.1)	72 (36.0)	215 (26.5)	
**MEDLIFE score, mean ± SD**	15.93 ± 3.39 ^a^	15.22 ± 3.74 ^a^	17.95 ± 3.40 ^b^	15.97 ± 3.63 ^a^	16.28 ± 3.68	**<0.001**
**Medlife quartiles, n (%)**						
Quartile 1 (≤14)	71 (35.3) ^a^	87 (43.1) ^a^	26 (12.4) ^b^	72 (36.0) ^a^	256 (31.5)	**<0.001**
Quartile 2 (15–16)	37 (18.4)	41 (20.3)	37 (17.7)	41 (20.5)	156 (19.2)	
Quartile 3 (17–19)	63 (31.3) ^ab^	48 (23.8) ^b^	75 (35.9) ^a^	52 (26.0) ^ab^	238 (29.3)	
Quartile 4 (≥20)	30 (14.9) ^a^	26 (12.9) ^a^	71 (34.0) ^b^	35 (17.5) ^a^	162 (20.0)	
**For Children/Adolescents**	**Türkiye (n = 124)**	**Italy (n = 101)**	**Lebanon (n = 124)**	**Spain (n = 151)**	**Total (n = 500)**	***p***
**KIDMED score, mean ± SD**	6.29 ± 2.23	5.93 ± 2.44	5.93 ± 2.36	6.46 ± 2.47	6.18 ± 2.38	0.119
**KIDMED groups, n (%)**						
Poor (≤3)	12 (9.7)	16 (15.8)	13 (10.5)	16 (10.5)	57 (11.4)	0.535
Average (4–7)	73 (58.8)	61 (60.4)	79 (63.7)	86 (57.0)	299 (59.8)	
High (≥8)	39 (31.4)	24 (23.8)	32 (25.8)	49 (32.5)	144 (28.8)	

KIDMED: Mediterranean diet quality index; MEDAS: Mediterranean diet adherence screener; MedDiet: Mediterranean diet. Chi-square test. Kruskal–Wallis test. The ‘a’, ‘b’, and ‘c’ superscripts show the results of pairwise comparisons between countries; values with different letters were significantly different among groups. The bold values indicate statistically significant values (*p* < 0.05).

**Table 3:** Main categories of obstacles to and drivers of adherence to the Mediterranean diet of the sample population.

	Türkiye (n = 201)	Italy (n = 202)	Lebanon (n = 209)	Spain (n = 200)	Total (n = 812)	*p*
**DRIVERS**	**Mean ± SD**	**Mean ± SD**	**Mean ± SD**	**Mean ± SD**	**Mean ± SD**	
Health	4.33 ± 0.82 ^a^	4.30 ± 0.80 ^a^	3.93 ± 0.84 ^b^	4.05 ± 0.93 ^b^	4.15 ± 0.87	**<0.001**
Diet quality	4.49 ± 0.61 ^a^	4.46 ± 0.64 ^a^	4.12 ± 0.77 ^b^	4.16 ± 0.78 ^b^	4.31 ± 0.72	**<0.001**
Applicability	4.01 ± 1.08 ^ab^	4.28 ± 0.87 ^a^	3.89 ± 0.99 ^b^	3.99 ± 1.01 ^b^	4.04 ± 1.00	**<0.001**
Lifestyle	4.06 ± 0.77 ^ab^	4.12 ± 0.80 ^a^	3.90 ± 0.79 ^b^	3.98 ± 0.80 ^ab^	4.01 ± 0.79	**0.020**
Affordability	3.57 ± 1.13	3.41 ± 1.03	3.52 ± 0.96	3.33 ± 0.97	3.46 ± 1.02	0.094
Environmental factors	3.98 ± 0.77 ^a^	3.84 ± 0.84 ^ab^	3.66 ± 0.98 ^b^	3.61 ± 0.91 ^b^	3.77 ± 0.89	**<0.001**
**OBSTACLES**						
Health	2.18 ± 0.78	2.29 ± 0.78	2.27 ± 0.79	2.21 ± 0.87	2.24 ± 0.80	0.460
Lifestyle	2.28 ± 1.37	2.08 ± 1.19	2.10 ± 1.16	2.12 ± 1.12	2.15 ± 1.22	0.681
Affordability	2.47 ± 1.11 ^a^	2.29 ± 0.96 ^a^	2.72 ± 0.79 ^b^	2.62 ± 0.96 ^ab^	2.53 ± 0.97	**<0.001**

Kruskal–Wallis test. The ‘a’ and ‘b’ superscripts show the results of pairwise comparisons between countries; values with different letters were significantly different among groups. The bold values indicate statistically significant values (*p* < 0.05).

## Data Availability

The data presented in this study are available on request from the corresponding author. The data are not publicly available due to privacy.

## Supplementary Materials

The following supporting information can be downloaded at: https://www.mdpi.com/article/10.3390/nu17071157/s1. Supplementary File S1: Final questionnaire; Table S1: Obstacles to and drivers of the Mediterranean diet; Table S2: Comparison of family relationships by country; Table S3: The relationship between family relationships and adherence to the Mediterranean diet of children and adolescents; Table S4: Obstacles to and drivers of adherence to the Mediterranean diet of the sample population.

## Abbreviations

The following abbreviations are used in this manuscript:

BMI	Body mass index
KIDMED	Mediterranean diet quality index
MEDAS	Mediterranean diet adherence screener
MedDiet	Mediterranean diet
MEDLIFE	Mediterranean lifestyle index
PREDIMED	Prevención con Dieta Mediterránea
UNESCO	United Nations Educational, Scientific and Cultural Organization
