# Supplementary material for: Adherence to the Mediterranean Diet Among Families from Four Countries in the Mediterranean Basin
Source: Nutrients. 2025 Mar 27;17(7):1157. doi: 10.3390/nu17071157 (PMC11990228; doi:10.3390/nu17071157)
Supplement: Supplementary file 1 [file nutrients-17-01157-s001.zip › Supplementary File S1.pdf]

Volunteer Number:					
**A. SOCIODEMOGRAPHIC CHARACTERISTICS**					
**1.**	Age (Year of birth):				
**2.**	Gender:	1. Male		2. Female	
**3.**	Education Level:	1. Non-finished primary school		2. Primary school	3. Secondary school
		4. High school		5. Vocational school	6. University
		7. Post-graduate			
**4.**	Parent’s Working Status:	1. Both parents	2. Only father	3. Only mother	4. None of parents
**5.**	Occupation:	1. Managers		2. Academic laborer	3. Office worker
		4. Service worker (food service, cleaning service, personal service, and protective service)		5. Agriculture and forestry worker	6. Blue-collar worker
		7. Engine worker		8. Healthcare worker	9. Unskilled laborer
**6.**	Household income status:	1. Low *(Income less than expenses)*		2. Middle *(Equivalent to income and expenses)*	3. High *(Income more than expenses)*
**7.**	Marital status:	1. Single		2. Seperated/divorced	3. Married/with partners
**8.**	Types of Family:	1. Elementary family **A couple and their dependent children*		2. Extended family **A family which extends beyond the elementary family to include grandparents and other relatives*	
**9.**	Number of children:	1. None		2. One	
		3. Two		4. ≥Three	
**10.**	Ages of children:	1st child: …………		2nd child: …………..	3rd child: …………
		4rd child: …………		5th child: ……………	6th child: …………
**11.**	Who do you live with?	1. Alone		2. With family	
		3. With friend		4. Other	
**12.**	Body weight	…………… kg			
**13.**	Height	…………… cm			

**B. FAMILY RELATIONSHIPS**						
**1.**	Do all of your family living in your home eat breakfast together?	1. Always	2. 4 to 6 times per week		3. Less than two times	4. Only weekends
**2.**	Do all of your family living in your home eat dinner together?	1. Always	2. 4 to 6 times per week		3. Less than two times	4. Only weekends
**3.**	Does at least one parent accompany your child at dinner?	1. Always	2. 4 to 6 times per week		3. Less than two times	4. Only weekends
**4.**	Do you watch TV during family meal?	1. Always	2. 4 to 6 times per week		3. Less than two times	4. Only weekends
**5.**	Do you answer the phone during the family meal?	1. Always	2. Usually	3. Often	4. Sometimes	5. Never
**6.**	Do you allow your child watch TV during family meal?	1. Always	2. Usually	3. Often	4. Sometimes	5. Never
**7.**	Do you allow your child answer the phone during the family meal?	1. Always	2. Usually	3. Often	4. Sometimes	5. Never
**8.**	How often do you invite people at dinner or lunch for social events? (Number of invitation)	……………………………… week/month/year				

C. THE MEDITERRANEAN DIET ADHERENCE SCREENER (MEDAS)			
	**ITEM**	**1 POINT**	**0 POINT**
**1.**	Do you use olive oil as the principal source of fat for cooking?	Yes	No
**2.**	How much olive oil do you consume per day (including that used in frying, salads, meals eaten away from home, etc.)?	At least 4 table spoons	Less than 4 table spoons
**3.**	How many servings of vegetables do you consume per day?	At least 2	Less than 2
**4.**	How many pieces of fruit (including fresh-squeezed juice) do you consume per day?	At least 3	Less than 3
**5.**	How many servings of red meat, hamburger, or sausages do you consume per day?	Less than 1	At least 1
**6.**	How many servings (12 g) of butter, margarine, or cream do you consume per day?	Less than 1	At least 1
**7.**	How many carbonated and/or sugar-sweetened beverages do you consume per day?	Less than 1	At least 1
**8.**	Do you drink wine? How much do you consume per week?	At least seven cups	Less than seven cups
**9.**	How many servings of pulses do you consume per week?	At least 3	Less than 3
**10.**	How many servings of fish/seafood do you consume per week?	At least 3	Less than 3
**11.**	How many times do you consume commercial (not homemade) pastry such as cookies or cake per week?	Less than 2	At least 2
**12.**	How many times do you consume nuts per week?	At least 3	Less than 3
**13.**	Do you prefer to eat chicken, turkey or rabbit instead of beef, pork, hamburgers, or sausages?	Yes	No
**14.**	How many times per week do you consume boiled vegetables, pasta, rice, or other dishes with a sauce of tomato, garlic, onion, or leeks sautéed in olive oil?	At least 3	Less than 3

D. THE MEDITERRANEAN DIET QUALITY INDEX (KIDMED)			
	**ITEM**	**YES**	**NO**
**1.**	Takes a fruit or fruit juice every day.		
**2.**	Has a second fruit every day.		
**3.**	Has fresh or cooked vegetables regularly once a day.		
**4.**	Has fresh or cooked vegetables more than once a day.		
**5.**	Consumes fish regularly (at least 2–3/week).		
**6.**	Goes >1/ week to a fast food restaurant (hamburger).		
**7.**	Likes pulses and eats them >1/week		
**8.**	Consumes pasta or rice almost every day (5 or more per week).		
**9.**	Has cereals or grains (bread, etc) for breakfast.		
**10.**	Consumes nuts regularly (at least 2–3/week).		
**11.**	Uses olive oil at home.		
**12.**	Skips breakfast.		
**13.**	Has a dairy product for breakfast (yoghurt, milk, etc).		
**14.**	Has commercially baked goods or pastries for breakfast.		
**15.**	Takes two yoghurts and/or some cheese (40 g) daily.		
**16.**	Takes sweets and candy several times every day.		

E. MEDITERRANEAN LIFESTYLE (MEDLIFE) QUESTIONNAIRE		
**ITEMS**	**CRITERIA FOR 1 POINT***	**POINT**
**BLOCK 1: MEDITERRANEAN FOOD CONSUMPTION**		
**1.** How many serving of pastries do you consume per week? (candy (1s=1 unit or 50 g), chocolates (1 s=30gr), biscuits (1 s=4-6 units), turron (1s=40 g))	≤2 p/week	
**2.** How many servings of red meat do you consume per week? (Beef, pork, lamb (1 s=100-150g))	< 2 p/week	
**3.** How many serving of processed meat do you consume per week? (Ham (1 s=1 slice or 30 g), sausage, soft spicy sausage, bacon (1 s=50 g), hamburger (1 s=1 unit), liver (1 s=100-15 0g), pathe (1s=25g))	≤ 1 p/week	
**4.** How many eggs do you consume per week? (Eggs (1 egg))	2-4 p/week	
**5.** How many serving of legumes do you consume per week? (Lentils, beans, peas, chickpeas (1 s=1 plate or 150 g))	≥ 2/ week	
**6.** How many servings of white meat do you consume per week? (Poultry, rabbit (1 s=100-150 g))	2 p/week	
**7.** How many serving of fish or seafood portions do you consume per week? (White/oily fish (1 s=100-150 g), canned fish (1 s=1 can or 50 g), seafood (1 s=200g))	≥ 2/ week	
**8.** How many potatoes do you consume per week? (Roast/boiled potatoes, French fries (1 s=150-200 g))	≤ 3 p/week	
**9.** How many low fat dairy products do you consume per day? (Skimmed dairy milk (1s=200 mL milk, two yoghurts, 1 portion soft cheese))	2 p/day	
**10.** How many nuts and olives do you consume per day? (Walnuts, almonds, hazelnuts (1s=1 handful or 30 g), olives (1 s=10 units))	1-2 p/day	
**11.** How many times do you use herbs, spices and garnish for cooking per day? (Onion, garlic, herbs (parsley, oregano))	≥ 1 p/day	
**12.** How many pieces of fruit do you consume per day? (All fruit and fresh fruit-based juices (1 s=150-200g))	3-6 p/day	
**13.** How many serving of vegetables you consume per day? (All vegetables except potatoes (1 s=150-200 g))	≥ 2 p/day	
**14.** How many olive oil tablespoons do you consume per day (cooking or dress salad)? (Olive oil, virgin olive oil (1s=1Tablespoon))	≥ 3 p/day	
**15.** How many serving of cereals you consume per day? (White and whole-grain bread (1s=40 g), cereals (1s=1 plate rice, pasta or 40g breakfast cereals) and derivatives)	3-6 p/day	
**BLOCK 2: MEDITERRANEAN DIETARY HABITS**		
**16.** Do you drink more than 6 glasses of water or at least one cup of tea per day? (Water or tea (1 s=1 glass))	Yes	
**17.** Do you drink wine during the meals every day? (White/red wine (1 s=1 glass of wine))	1-2 s/day	
**18.** Do you limit salt in meals?	Yes	
**19.** Do you usually choose whole grain products? (bread, pasta, rice, breakfast cereals)	Yes	
**20.** Do you consume snacks 2 or less time per week? (potatoes chips, tortilla chips, popcorn (1 s=1 bag or 50 g))	Yes	
**21.** Do you usually limit nibbling between meals?	Yes	
**22.** Do you limit sugar in beverages? (including sugar-sweetened beverages)	Yes	
**23.** Do you prefer and consume seasonal and traditional local products, fresh and minimally processed food?	Yes	
**24.** Do you prefer and consume with moderation trying to choose small portion sizes?	Yes	
**BLOCK 3: PHYSICAL ACTIVITY, REST, SOCIAL HABITS AND CONVIVIALITY**		
**25.** Do you engage in physical activity (>150min/week or 30 min/day)? (jogging, walk at a fast pace, dance, aerobics, gardening)	Yes	
**26.** Do you sleep siesta/nap?	Yes	
**27.** How many hours do you sleep a day? (During weekdays)	6-8 hour/day	
**28.** How many hours do you spend watching TV per day? (During weekdays)	≤1hour/day	
**29.** How many hours do you spend going out with friends during the free time (e.g. weekends)?	≥2hour/weekend	
**30.** How many hours do you practice team sports per week?	≥2hour/week	
**31.** How many time do you spend having lunch during weekdays?	≥ 20 minutes	
**32.** Do you usually eat in company (with family, friends, and colleagues)?	Yes	
*** 0 points if these criteria are no met s = serving**		

F. ATTITUDES TOWARDS MEDITERRANEAN DIET<br><br>Mediterranian diet: The principal aspects of this diet include proportionally high consumption of olive oil, legumes, unrefined cereals, fruits, and vegetables, moderate to high consumption of fish, moderate consumption of dairy products (mostly as cheese and yogurt), and low consumption of red and processed meats.<br><br>Please rate the factors you believe that encourage (DRIVERS) and hinder (OBSTACLES) you and your family to follow a Mediterranean diet (MD).							
**DRIVERS**							
	**ITEMS**		1<br>Not at all true for me				5<br>Very true for me
**HEALTH**	**1.**	How knowledgeable do you feel about the topic of sustainability?	1□	2□	3□	4□	5□
	**2.**	MD lowers LDL (bad) cholesterol levels.	1□	2□	3□	4□	5□
	**3.**	MD reduces the risk of cardiovascular disease, diabetes, mental illness, depression, cancer, and obesity.	1□	2□	3□	4□	5□
**DIET QUALITY**	**4.**	MD includes healthier and more nutritious foods.	1□	2□	3□	4□	5□
	**5.**	MD is definied higher fruit and vegetable consumption and lower red meat consumption.	1□	2□	3□	4□	5□
	**6.**	MD includes more beneficial oils for health.	1□	2□	3□	4□	5□
**APPLICABILITY**	**7.**	MD is tastier and more sustainable than other types of diets.	1□	2□	3□	4□	5□
**LIFESTYLE**	**8.**	MD increases consumption of homemade foods.	1□	2□	3□	4□	5□
	**9.**	MD includes more unprocessed and additive-free foods.	1□	2□	3□	4□	5□
	**10.**	MD is associated with higher socialization and family relationships.	1□	2□	3□	4□	5□
**AFFORDABILITY**	**11.**	Food access is easier in MD.	1□	2□	3□	4□	5□
	**12.**	MD contains lower-priced foods.	1□	2□	3□	4□	5□
**ENVIRONMENT**	**13.**	MD has a positive effect on the environment.	1□	2□	3□	4□	5□
	**14.**	MD reduces human impact on the environment.	1□	2□	3□	4□	5□
	**15.**	MD is associated with better carbon footprint.	1□	2□	3□	4□	5□
	**16.**	MD contains more local foods.	1□	2□	3□	4□	5□
**OBSTACLES**							
**HEALTH**	**17.**	MD contains more allergenic foods.	1□	2□	3□	4□	5□
	**18.**	MD is not applicable for vegans.	1□	2□	3□	4□	5□
	**19.**	Food variety in MD is insufficient.	1□	2□	3□	4□	5□
	**20.**	Preparing meals suitable for MD is difficult and time-consuming.	1□	2□	3□	4□	5□
	**21.**	MD is restrictive.	1□	2□	3□	4□	5□
	**22.**	MD contains unpleasant-tasting foods.	1□	2□	3□	4□	5□
	**23.**	It is difficult to diversify food recipes in MD.	1□	2□	3□	4□	5□
**LIFESTYLE**	**24.**	Following MD is difficult due to conflict with cultural habits/beliefs/norms.	1□	2□	3□	4□	5□
**AFFORDABILITY**	**25.**	MD contains high-priced foods.	1□	2□	3□	4□	5□
	**26.**	There are limited options in shops for foods in MD.	1□	2□	3□	4□	5□
	**27.**	There are limited options in restaurants for foods in MD.	1□	2□	3□	4□	5□

MD: Mediterranian diet
