# Supplementary material for: Adherence to the Mediterranean Diet Among Families from Four Countries in the Mediterranean Basin
Source: Nutrients. 2025 Mar 27;17(7):1157. doi: 10.3390/nu17071157 (PMC11990228; doi:10.3390/nu17071157)
Supplement: Supplementary file 1 [file nutrients-17-01157-s001.zip › Supplementary Tables S1-S4.pdf]

Adherence to the Mediterranean Diet Among Families from Four Countries in the Mediterranean Basin

Table S1. Obstacles and drivers to the Mediterranean diet.

**DRIVERS**						
	**ITEMS**	**1 Not at all true for me**				**5 Very true for me**
**1**	MedDiet has a positive effect on cholesterol.	1□	2□	3□	4□	5□
**2**	MedDiet lowers LDL (bad) cholesterol levels.	1□	2□	3□	4□	5□
**3**	MedDiet reduces the risk of cardiovascular disease, diabetes, mental illness, depression, cancer, and obesity.	1□	2□	3□	4□	5□
**4**	MedDiet includes healthier and more nutritious foods.	1□	2□	3□	4□	5□
**5**	MedDiet is definied higher fruit and vegetable consumption and lower red meat consumption.	1□	2□	3□	4□	5□
**6**	MedDiet includes more beneficial oils for health.	1□	2□	3□	4□	5□
**7**	MedDiet is tastier and more sustainable than other types of diets.	1□	2□	3□	4□	5□
**8**	MedDiet increases consumption of homemade foods.	1□	2□	3□	4□	5□
**9**	MedDiet includes more unprocessed and additive-free foods.	1□	2□	3□	4□	5□
**10**	MedDiet is associated with higher socialization and family relationships.	1□	2□	3□	4□	5□
**11**	Food access is easier in MedDiet.	1□	2□	3□	4□	5□
**12**	MedDiet contains lower-priced foods.	1□	2□	3□	4□	5□
**13**	MedDiet has a positive effect on the environment.	1□	2□	3□	4□	5□
**14**	MedDiet reduces human impact on the environment.	1□	2□	3□	4□	5□
**15**	MedDiet is associated with better carbon footprint.	1□	2□	3□	4□	5□
**16**	MedDiet contains more local foods.	1□	2□	3□	4□	5□
**OBSTACLES**						
**17**	MedDiet contains more allergenic foods.	1□	2□	3□	4□	5□

**18**	MedDiet is not applicable for vegans.	1□	2□	3□	4□	5□
**19**	Food variety in MedDiet is insufficient.	1□	2□	3□	4□	5□
**20**	Preparing meals suitable for MedDiet is difficult and time-consuming.	1□	2□	3□	4□	5□
**21**	MedDiet is restrictive.	1□	2□	3□	4□	5□
**22**	MedDiet contains unpleasant-tasting foods.	1□	2□	3□	4□	5□
**23**	It is difficult to diversify food recipes in MedDiet.	1□	2□	3□	4□	5□
**24**	Following MedDiet is difficult due to conflict with cultural habits/beliefs/norms.	1□	2□	3□	4□	5□
**25**	MedDiet contains high-priced foods.	1□	2□	3□	4□	5□
**26**	There are limited options in shops for foods in MedDiet.	1□	2□	3□	4□	5□
**27**	There are limited options in restaurants for foods in MedDiet.	1□	2□	3□	4□	5□

Table S2. Comparison of family relationships by country.

	Türkiye (n=201)	Italy (n=202)	Lebanon (n=209)	Spain (n=200)	Total (n=812)	*p*
	**n (%)**	**n (%)**	**n (%)**	**n (%)**	**n (%)**	
**Do all of your family living in your home eat breakfast together?**						
Always	69 (34.3)^{a}	42 (20.8)^{bc}	70 (33.5)^{a}	40 (20.0)^{b}	221 (27.2)	
4 to 6 times per week	18 (9.0)^{a}	32 (15.8)^{a}	30 (14.4)^{a}	30 (15.0)^{a}	110 (13.5)	**0.006**
Less than two times	36 (17.9)^{a}	40 (19.8)^{a}	41 (19.6)^{a}	45 (22.5)^{a}	162 (20.0)	
Only weekends	78 (38.8)^{a}	88 (43.6)^{a}	68 (32.5)^{a}	85 (42.5)^{a}	319 (39.3)	
**Do all of your family living in your home eat dinner together?**						
Always	137 (68.2)^{a}	123 (60.9)^{ab}	102 (48.8)^{b}	119 (59.5)^{ab}	481 (59.2)	
4 to 6 times per week	28 (13.9)^{ab}	63 (31.2)^{c}	22 (10.5)^{b}	48 (24.0)^{ac}	161 (19.8)	**<0.001**
Less than two times	29 (14.4)^{ab}	8 (4.0)^{c}	32 (15.3)^{b}	14 (7.0)^{ac}	83 (10.2)	
Only weekends	7 (3.5)^{a}	8 (4.0)^{a}	53 (25.4)^{b}	19 (9.5)^{a}	87 (10.7)	
**Does at least one parent accompany your child at dinner?**						
Always	127 (87.6)^{a}	124 (87.9)^{a}	81 (51.3)^{b}	121 (80.1)^{a}	453 (76.1)	
4 to 6 times per week	6 (4.1)^{a}	10 (7.1)^{a}	9 (5.7)^{a}	19 (12.6)^{a}	44 (7.4)	**<0.001**
Less than two times	9 (6.2)^{a}	3 (2.1)^{a}	43 (27.2)^{b}	5 (3.3)^{a}	60 (10.1)	
Only weekends	3 (2.1)^{a}	4 (2.8)^{a}	25 (15.8)^{b}	6 (4.0)^{a}	38 (6.4)	
**Do you watch TV during the family meal?**						
Always	43 (21.4)^{a}	90 (44.6)^{b}	36 (17.2)^{a}	74 (37.0)^{b}	243 (29.9)	
4 to 6 times per week	30 (14.9)^{a}	36 (17.8)^{a}	22 (10.5)^{a}	35 (17.5)^{a}	123 (15.1)	**<0.001**
Less than two times	103 (51.2)^{a}	52 (25.7)^{b}	103 (49.3)^{a}	49 (24.5)^{b}	307 (37.8)	
Only weekends	25 (12.4)^{a}	24 (11.9)^{a}	48 (23.0)^{b}	42 (21.0)^{ab}	139 (17.1)	
**Do you answer the phone during the family meal?**						
Always	35 (17.4)^{a}	17 (8.4)^{b}	49 (23.4)^{a}	14 (7.0)^{b}	115 (14.2)	
Usually	40 (19.9)^{a}	21 (10.4)^{b}	57 (27.3)^{ac}	63 (31.5)^{c}	181 (22.3)	
Often	15 (7.5)^{abc}	6 (3.0)^{c}	29 (13.9)^{b}	8 (4.0)^{ac}	58 (7.1)	**<0.001**
Sometimes	84 (41.8)^{ab}	110 (54.5)^{b}	44 (21.1)^{c}	80 (40.0)^{a}	318 (39.2)	
Never	27 (13.4)^{a}	48 (23.8)^{b}	30 (14.4)^{ab}	35 (17.5)^{ab}	140 (17.2)	
**Do you allow your child to watch TV during family meals?**						
Always	19 (13.2) ^{a}	39 (28.5)^{b}	24 (15.2)^{a}	36 (23.8)^{ab}	118 (20.0)	
Usually	17 (11.8) ^{a}	16 (11.7)^{a}	31 (19.6)^{ab}	48 (31.8)^{b}	112 (19.0)	
Often	5 (3.5)	19 (13.9)^{b}	20 (12.7)^{b}	12 (7.9)^{ab}	56 (9.5)	**<0.001**
Sometimes	55 (38.2)^{a}	31 (22.6)^{b}	48 (30.4)^{ab}	37 (24.5)^{ab}	171 (29.0)	

Never	48 (33.3)[a]	32 (23.4)[ab]	35 (22.2)[ab]	18 (11.9)[b]	133 (22.5)	
**Do you allow your child to answer the phone during the family meal?**						
Always	14 (9.7)[a]	15 (10.9)[a]	37 (23.4)[b]	6 (4.0)[a]	72 (12.2)	
Usually	15 (10.4)[ab]	9 (6.6)[b]	28 (17.7)[a]	21 (13.9)[ab]	73 (12.4)	
Often	9 (6.3)[ab]	7 (5.1)[ab]	22 (13.9)[b]	3 (2.0)[a]	41 (6.9)	**<0.001**
Sometimes	49 (34.0)[a]	35 (25.5)[a]	44 (27.8)[a]	48 (31.8)[a]	176 (29.8)	
Never	57 (39.6)[a]	71 (51.8)[a]	27 (17.1)[b]	73 (48.3)[a]	228 (38.6)	
**How often do you invite dinner or lunch at social events? (Frequency of invitation)**						
Daily	1 (0.5)[a]	0 (0.0)[a]	1 (0.5)[a]	20 (10.0)[b]	22 (2.7)	
Weekly	63 (31.3)[a]	81(40.1)[a]	2 (1.0)[b]	13 (6.5)[c]	159 (19.6)	
Monthly	100 (49.8)[ab]	86 (42.6)[bc]	128 (61.2)[a]	64 (32.0)[c]	378 (46.6)	**<0.001**
Yearly	37 (18.4)[a]	19 (9.4)[a]	78 (37.3)[b]	103 (51.5)[c]	237 (29.2)	
Never	0 (0.0)[a]	16 (7.9)[b]	0 (0.0)[a]	0 (0.0)[a]	16 (2.0)	

*Pearson Chi-Square and Fisher-Freeman-Halton tests were used. The Bonferroni test was used for pairwise comparisons. The "a", "b", and "c" superscripts show the results of pairwise comparisons between countries; values with unlike letters were significantly different among groups.

Table S3. The relationship between family relationships and adherence to the Mediterranean diet of children and adolescents.

Family Relationships	KIDMED	
	r	*p*
Frequency of having breakfast together among all family members living at home	0.093	**0.022**
Frequency of having dinner together among all family members living at home	-0.002	0.958
Frequency of at least one parent accompanying the child at dinner	0.020	0.623
Frequency of watching TV during family meal	-0.092	**0.025**
Frequency of answering the phone during the family meal	0.080	0.051
Frequency of allowing children to watch TV during family meal	-0.141	**0.001**
Frequency of allowing children to answer the phone during family meal	0.088	0.051
Frequency of invitation dinner or lunch at social events	0.071	0.081
Number of invitations for dinner or lunch at social events	-0.016	0.700

**r:* Spearman's rho correlation coefficient.

Table S4. Obstacles and drivers to adherence to the Mediterranean diet of the sample population.

Country		Items	1 Not at all true for me		2		3		4		5 Very true for me		Mean	SD
			n	%	n	%	n	%	n	%	n	%		
Türkiye (n = 201)	Drivers	6	0	0.0%	0	0.0%	19	9.5%	44	21.9%	138	68.7%	4.59	0.66
		4	2	1.0%	0	0.0%	25	12.4%	38	18.9%	136	67.7%	4.52	0.79
		3	3	1.5%	2	1.0%	30	14.9%	46	22.9%	120	59.7%	4.38	0.88
		5	0	0.0%	3	1.5%	40	19.9%	39	19.4%	119	59.2%	4.36	0.85
		16	1	0.5%	3	1.5%	35	17.4%	49	24.4%	113	56.2%	4.34	0.85
		2	3	1.5%	4	2.0%	36	17.9%	39	19.4%	119	59.2%	4.33	0.94
		8	4	2.0%	3	1.5%	37	18.4%	38	18.9%	119	59.2%	4.32	0.96
		9	5	2.5%	5	2.5%	34	16.9%	38	18.9%	119	59.2%	4.30	1.00
		1	4	2.0%	4	2.0%	35	17.4%	44	21.9%	114	56.7%	4.29	0.96
		13	4	2.0%	1	0.5%	62	30.8%	39	19.4%	95	47.3%	4.09	0.99
		7	6	3.0%	8	4.0%	55	27.4%	40	19.9%	92	45.8%	4.01	1.08
		11	9	4.5%	18	9.0%	54	26.9%	35	17.4%	85	42.3%	3.84	1.20
		15	0	0.0%	12	6.0%	86	42.8%	35	17.4%	68	33.8%	3.79	0.98
		14	11	5.5%	8	4.0%	82	40.8%	31	15.4%	69	34.3%	3.69	1.15
		10	16	8.0%	10	5.0%	79	39.3%	37	18.4%	59	29.4%	3.56	1.19

		12	30	14.9%	27	13.4%	55	27.4%	29	14.4%	60	29.9%	3.31	1.41
		25	63	31.3%	26	12.9%	48	23.9%	33	16.4%	31	15.4%	2.72	1.45
		18	70	34.8%	28	13.9%	56	27.9%	12	6.0%	35	17.4%	2.57	1.45
		27	71	35.3%	27	13.4%	46	22.9%	33	16.4%	24	11.9%	2.56	1.42
		17	73	36.3%	30	14.9%	63	31.3%	18	9.0%	17	8.5%	2.38	1.29
		20	91	45.3%	23	11.4%	46	22.9%	18	9.0%	23	11.4%	2.30	1.41
	**Obstacles**	24	90	44.8%	21	10.4%	54	26.9%	15	7.5%	21	10.4%	2.28	1.37
		21	87	43.3%	28	13.9%	54	26.9%	17	8.5%	15	7.5%	2.23	1.29
		26	99	49.3%	29	14.4%	36	17.9%	20	10.0%	17	8.5%	2.14	1.35
		22	110	54.7%	23	11.4%	39	19.4%	16	8.0%	13	6.5%	2.00	1.28
		23	105	52.2%	35	17.4%	32	15.9%	14	7.0%	15	7.5%	2.00	1.28
		19	121	60.2%	30	14.9%	34	16.9%	9	4.5%	7	3.5%	1.76	1.10
		5	2	1.0%	3	1.5%	19	9.4%	46	22.8%	132	65.3%	4.50	0.81
		4	1	0.5%	2	1.0%	16	7.9%	61	30.2%	122	60.4%	4.49	0.73
		6	1	0.5%	3	1.5%	24	11.9%	60	29.7%	114	56.4%	4.40	0.79
**Italy (n=202)**	**Drivers**	1	3	1.5%	3	1.5%	26	12.9%	55	27.2%	115	56.9%	4.37	0.87
		3	4	2.0%	4	2.0%	27	13.4%	58	28.7%	109	54.0%	4.31	0.92
		7	2	1.0%	6	3.0%	26	12.9%	67	33.2%	101	50.0%	4.28	0.87
		9	1	0.5%	11	5.4%	23	11.4%	63	31.2%	104	51.5%	4.28	0.90

	2	5	2.5%	7	3.5%	27	13.4%	63	31.2%	100	49.5%	4.22	0.97
	8	4	2.0%	8	4.0%	31	15.3%	66	32.7%	93	46.0%	4.17	0.96
	16	4	2.0%	10	5.0%	37	18.3%	66	32.7%	85	42.1%	4.08	0.99
	10	6	3.0%	20	9.9%	41	20.3%	56	27.7%	79	39.1%	3.90	1.12
	13	4	2.0%	10	5.0%	58	28.7%	64	31.7%	66	32.7%	3.88	0.99
	14	5	2.5%	11	5.4%	61	30.2%	66	32.7%	59	29.2%	3.81	1.00
	11	10	5.0%	15	7.4%	62	30.7%	61	30.2%	54	26.7%	3.66	1.10
	15	6	3.0%	13	6.4%	84	41.6%	55	27.2%	44	21.8%	3.58	1.00
	12	21	10.4%	33	16.3%	71	35.1%	47	23.3%	30	14.9%	3.16	1.18
	19	47	23.3%	32	15.8%	46	22.8%	28	13.9%	49	24.3%	3.00	1.49
	25	47	23.3%	42	20.8%	69	34.2%	31	15.3%	13	6.4%	2.61	1.18
	20	52	25.7%	49	24.3%	53	26.2%	30	14.9%	18	8.9%	2.57	1.26
	17	42	20.8%	62	30.7%	56	27.7%	27	13.4%	15	7.4%	2.56	1.18
**Obstacles**	18	76	37.6%	40	19.8%	35	17.3%	21	10.4%	30	14.9%	2.45	1.45
	27	70	34.7%	54	26.7%	51	25.2%	17	8.4%	10	5.0%	2.22	1.16
	24	87	43.1%	50	24.8%	37	18.3%	18	8.9%	10	5.0%	2.08	1.19
	26	86	42.6%	51	25.2%	45	22.3%	11	5.4%	9	4.5%	2.04	1.13
	21	92	45.5%	53	26.2%	32	15.8%	15	7.4%	10	5.0%	2.00	1.17
	23	108	53.5%	37	18.3%	35	17.3%	14	6.9%	8	4.0%	1.90	1.16

		22	143	70.8%	31	15.3%	13	6.4%	8	4.0%	7	3.5%	1.54	1.02
Lebanon (n=209)	Drivers	4	3	1.4%	9	4.3%	26	12.4%	66	31.6%	105	50.2%	4.25	0.93
		8	3	1.4%	8	3.8%	23	11.0%	81	38.8%	94	45.0%	4.22	0.89
		9	12	5.7%	8	3.8%	27	12.9%	55	26.3%	107	51.2%	4.13	1.14
		5	4	1.9%	16	7.7%	26	12.4%	82	39.2%	81	38.8%	4.05	1.00
		6	5	2.4%	8	3.8%	30	14.4%	95	45.5%	71	34.0%	4.05	0.92
		3	9	4.3%	11	5.3%	26	12.4%	87	41.6%	76	36.4%	4.00	1.04
		1	8	3.8%	11	5.3%	41	19.6%	82	39.2%	67	32.1%	3.90	1.03
		7	4	1.9%	16	7.7%	42	20.1%	83	39.7%	64	30.6%	3.89	0.99
		2	6	2.9%	14	6.7%	33	15.9%	99	47.6%	56	26.9%	3.89	0.97
		16	9	4.3%	21	10.0%	50	23.9%	52	24.9%	77	36.8%	3.80	1.17
		13	15	7.2%	23	11.0%	34	16.3%	73	34.9%	64	30.6%	3.71	1.22
		11	7	3.3%	23	11.0%	53	25.4%	71	34.0%	55	26.3%	3.69	1.08
		15	6	2.9%	28	13.4%	62	29.7%	69	33.0%	44	21.1%	3.56	1.05
		14	20	9.6%	26	12.4%	40	19.1%	64	30.6%	59	28.2%	3.56	1.28
		12	17	8.1%	40	19.1%	52	24.9%	54	25.8%	46	22.0%	3.34	1.24
		10	28	13.4%	34	16.3%	35	16.7%	64	30.6%	48	23.0%	3.33	1.35
	Obstacles	25	38	18.2%	44	21.1%	50	23.9%	53	25.4%	24	11.5%	2.91	1.28
		27	31	14.8%	65	31.1%	64	30.6%	34	16.3%	15	7.2%	2.70	1.13

		26	64	30.6%	32	15.3%	56	26.8%	47	22.5%	10	4.8%	2.56	1.27
		20	58	27.8%	59	28.2%	44	21.1%	37	17.7%	11	5.3%	2.44	1.22
		23	67	32.2%	41	19.7%	51	24.5%	44	21.2%	5	2.4%	2.42	1.21
		18	67	32.1%	61	29.2%	42	20.1%	29	13.9%	10	4.8%	2.30	1.19
		21	71	34.0%	57	27.3%	40	19.1%	31	14.8%	10	4.8%	2.29	1.22
		19	79	37.8%	54	25.8%	38	18.2%	28	13.4%	10	4.8%	2.22	1.22
		22	78	37.3%	57	27.3%	46	22.0%	22	10.5%	6	2.9%	2.14	1.12
		24	86	41.1%	55	26.3%	35	16.7%	27	12.9%	6	2.9%	2.10	1.16
		17	80	38.3%	61	29.2%	44	21.1%	21	10.0%	3	1.4%	2.07	1.06
**Spain (n=200)**	**Drivers**	4	1	0.5%	6	3.0%	37	18.5%	58	29.0%	98	49.0%	4.23	0.89
		9	2	1.0%	3	1.5%	39	19.5%	69	34.5%	87	43.5%	4.18	0.87
		8	3	1.5%	5	2.5%	38	19.0%	67	33.5%	87	43.5%	4.15	0.92
		5	3	1.5%	3	1.5%	41	20.5%	73	36.5%	80	40.0%	4.12	0.89
		3	2	1.0%	8	4.0%	40	20.0%	65	32.5%	85	42.5%	4.12	0.93
		6	2	1.0%	4	2.0%	47	23.5%	63	31.5%	84	42.0%	4.12	0.90
		2	9	4.5%	6	3.0%	43	21.5%	57	28.5%	85	42.5%	4.02	1.08
		1	9	4.5%	4	2.0%	50	25.0%	50	25.0%	87	43.5%	4.01	1.08
		7	4	2.0%	7	3.5%	58	29.0%	50	25.0%	81	40.5%	3.99	1.01
		16	3	1.5%	13	6.5%	53	26.5%	59	29.5%	72	36.0%	3.92	1.01

		11	8	4.0%	12	6.0%	68	34.0%	60	30.0%	52	26.0%	3.68	1.05
		13	5	2.5%	15	7.5%	75	37.5%	53	26.5%	52	26.0%	3.66	1.02
		10	13	6.5%	14	7.0%	66	33.0%	51	25.5%	56	28.0%	3.62	1.15
		14	9	4.5%	19	9.5%	85	42.5%	42	21.0%	45	22.5%	3.48	1.08
		15	9	4.5%	21	10.5%	87	43.5%	47	23.5%	36	18.0%	3.40	1.04
		12	26	13.0%	34	17.0%	81	40.5%	36	18.0%	23	11.5%	2.98	1.16
		25	27	13.5%	28	14.0%	73	36.5%	53	26.5%	19	9.5%	3.05	1.15
		20	56	28.0%	40	20.0%	60	30.0%	33	16.5%	11	5.5%	2.52	1.22
		27	54	27.0%	34	17.0%	83	41.5%	19	9.5%	10	5.0%	2.49	1.13
		26	64	32.0%	46	23.0%	58	29.0%	26	13.0%	6	3.0%	2.32	1.14
		23	68	34.0%	43	21.5%	60	30.0%	20	10.0%	9	4.5%	2.30	1.17
	**Obstacles**	18	78	39.0%	38	19.0%	58	29.0%	16	8.0%	10	5.0%	2.21	1.19
		17	76	38.0%	35	17.5%	69	34.5%	14	7.0%	6	3.0%	2.20	1.11
		21	75	37.5%	42	21.0%	59	29.5%	17	8.5%	7	3.5%	2.20	1.14
		19	84	42.0%	38	19.0%	54	27.0%	15	7.5%	9	4.5%	2.14	1.18
		24	79	39.5%	47	23.5%	52	26.0%	15	7.5%	7	3.5%	2.12	1.12
		22	99	49.5%	42	21.0%	40	20.0%	14	7.0%	5	2.5%	1.92	1.10
**Total (n = 812)**	**Drivers**	4	7	0.9%	17	2.1%	104	12.8%	223	27.5%	461	56.8%	4.37	0.85
		6	8	1.0%	15	1.8%	120	14.8%	262	32.3%	407	50.1%	4.29	0.85

	5	9	1.1%	25	3.1%	126	15.5%	240	29.6%	412	50.7%	4.26	0.91
	9	20	2.5%	27	3.3%	123	15.1%	225	27.7%	417	51.4%	4.22	0.99
	8	14	1.7%	24	3.0%	129	15.9%	252	31.0%	393	48.4%	4.21	0.93
	3	18	2.2%	25	3.1%	123	15.1%	256	31.5%	390	48.0%	4.20	0.96
	1	24	3.0%	22	2.7%	152	18.7%	231	28.4%	383	47.2%	4.14	1.01
	2	23	2.8%	31	3.8%	139	17.1%	258	31.8%	360	44.4%	4.11	1.01
	7	16	2.0%	37	4.6%	181	22.3%	240	29.6%	338	41.6%	4.04	1.00
	16	17	2.1%	47	5.8%	175	21.6%	226	27.8%	347	42.7%	4.03	1.03
	13	28	3.4%	49	6.0%	229	28.2%	229	28.2%	277	34.1%	3.83	1.07
	11	34	4.2%	68	8.4%	237	29.2%	227	28.0%	246	30.3%	3.72	1.11
	14	45	5.5%	64	7.9%	268	33.0%	203	25.0%	232	28.6%	3.63	1.14
	10	63	7.8%	78	9.6%	221	27.2%	208	25.6%	242	29.8%	3.60	1.22
	15	21	2.6%	74	9.1%	319	39.3%	206	25.4%	192	23.6%	3.58	1.03
	12	94	11.6%	134	16.5%	259	31.9%	166	20.4%	159	19.6%	3.20	1.26
	25	175	21.6%	140	17.2%	240	29.6%	170	20.9%	87	10.7%	2.82	1.28
	27	226	27.8%	180	22.2%	244	30.0%	103	12.7%	59	7.3%	2.49	1.22
**Obstacles**	20	257	31.7%	171	21.1%	203	25.0%	118	14.5%	63	7.8%	2.46	1.28
	18	291	35.8%	167	20.6%	191	23.5%	78	9.6%	85	10.5%	2.38	1.33
	17	271	33.4%	188	23.2%	232	28.6%	80	9.9%	41	5.0%	2.30	1.17

19	331	40.8%	154	19.0%	172	21.2%	80	9.9%	75	9.2%	2.28	1.33
26	313	38.5%	158	19.5%	195	24.0%	104	12.8%	42	5.2%	2.27	1.24
21	325	40.0%	180	22.2%	185	22.8%	80	9.9%	42	5.2%	2.18	1.21
23	348	42.9%	156	19.2%	178	21.9%	92	11.3%	37	4.6%	2.15	1.22
24	342	42.1%	173	21.3%	178	21.9%	75	9.2%	44	5.4%	2.15	1.22
22	430	53.0%	153	18.8%	138	17.0%	60	7.4%	31	3.8%	1.90	1.15

*Number codes indicate the factors (items) belonging to the drivers and barriers affecting adherence to the MedDiet. The drivers and obstacles corresponding to each code are given in Table S1.
